# The Role of Silicon During Solidification Process of Cast Al-Si-Mg Alloys

**DOI:** 10.3390/ma18215033

**Published:** 2025-11-05

**Authors:** Aleksandra Patarić, Mile Djurdjevic, Srecko Manasijevic, Srecko Stopic, Marija Mihailović

**Affiliations:** 1 Institute of Chemistry, Technology and Metallurgy, National Institute of the Republic of Serbia, University of Belgrade, Njegoševa 12, 11000 Belgrade, Serbia; aleksandra.pataric@ihtm.bg.ac.rs; 2 Department of Material Science and Technology, University of Applied Sciences Upper Austria, Roseggerstraße 15, 4600 Wels, Austria; miledjurdjevic@yahoo.ca; 3 Lola Institute Ltd., Kneza Viselava 70a, 11030 Belgrade, Serbia; srecko.manasijevic@li.rs; 4 IME Process Metallurgy and Metal Recycling, RWTH Aachen University, Intzestrasse 3, 52056 Aachen, Germany; sstopic@metallurgie.rwth-aachen.de

**Keywords:** feeding ability, silicon, cooling curve, Al-Si-Mg alloy, solidification temperatures

## Abstract

Hypoeutectic Al-Si-Mg alloys are among the most widely used casting materials in the automotive and aerospace industries due to their low density, high strength-to-weight ratio, corrosion resistance, and good castability. A critical challenge during solidification is shrinkage porosity, which arises from insufficient feeding and significantly reduces casting reliability. While the role of silicon in altering the phase diagram of Al-Si-Mg alloys is well-understood, its direct impact on feeding behavior has not been previously quantified in detail. In this study, the influence of silicon content (5–9 wt.%) on feeding ability was systematically investigated using thermal analysis (TA). By analyzing cooling curves, first derivatives, and ΔT curves, key solidification temperatures, including the liquidus, dendrite coherency, rigidity, and solidus, were precisely identified. For the first time, these data were used to quantitatively define all five feeding regions (liquid, mass, interdendritic, burst, and solid feeding) as a function of silicon content. The results demonstrate that increasing the Si content decreases the liquidus and dendrite coherency temperatures, raises the rigidity and solidus temperatures, and shortens the overall feeding ranges, particularly the interdendritic region (~32 °C reduction). This novel TA-based quantification of feeding regions provides insights that extend beyond classical phase diagram interpretation. The findings confirm that higher Si contents improve feeding ability by narrowing the freezing range and reducing the risk of porosity, while also providing a unique dataset for validating casting simulation software. The results also confirm that increasing the silicon content enhances feeding ability and improves castability by narrowing the freezing range and promoting more uniform solidification in Al-Si-Mg alloys. The study therefore bridges fundamental solidification science with industrial practice, supporting improved alloy design and defect control in Al-Si-Mg castings.

## 1. Introduction

Al-Si-Mg casting parts are well-known for their excellent mechanical properties and lighter weight than steel components. Hypoeutectic Al-Si-Mg alloys exhibit high strength, satisfactory tensile and fatigue performance, good corrosion resistance, fluidity, and low thermal expansion. Due to these attributes, combined with their reduced weight, these alloys are extensively used in the automotive, aerospace, construction, and other engineering industries. However, during the solidification of aluminum alloys, certain defects are unavoidable [1]. Among these, shrinkage porosity is caused by volume reduction during cooling and solidification, which significantly reduces the casting quality and increases production costs.

The addition of silicon (Si) and magnesium (Mg) to an aluminum matrix is a well-established practice in the development of Al-Si-Mg alloys. Silicon improves castability by lowering the melting temperature, increasing fluidity, and reducing shrinkage, while also enhancing wear resistance and hardness through the formation of hard Si particles. Magnesium, on the other hand, provides solid-solution strengthening and enables precipitation hardening when combined with silicon to form Mg_2_Si, significantly increasing the alloy’s strength. Together, these elements not only improve the mechanical properties, but also enhance corrosion resistance.

Depending on their composition, hypoeutectic Al-Si alloys exhibit shrinkage in the 4–8% range [2]. To moderate these volume deficits, recent research [3,4,5,6,7,8,9] has focused on improving feeding mechanisms and analyzing the influence of alloying elements on characteristic solidification temperatures.

**Figure 1:**
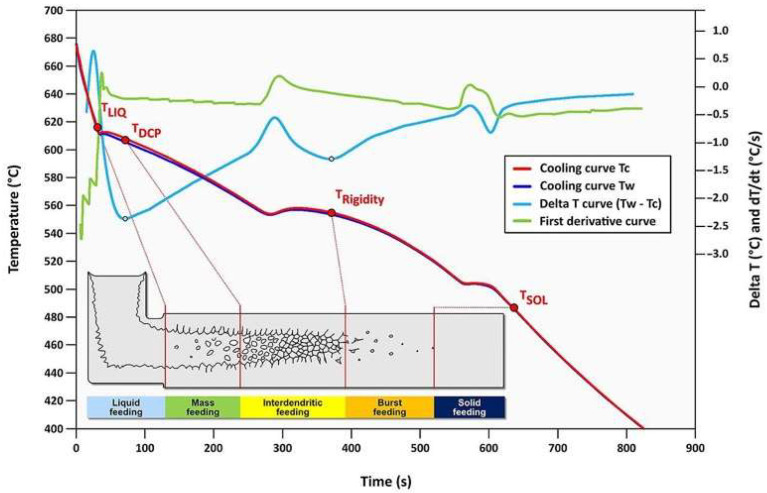
Schematic diagram of the five feeding mechanisms with appropriate specific solidification temperature.

Campbell [3,4] outlined five primary feeding mechanisms occurring during the solidification of aluminum cast alloys: liquid feeding, mass feeding, interdendritic feeding, burst feeding, and solid feeding. These mechanisms are delineated by specific solidification temperatures, namely the liquidus, dendrite coherency, rigidity, and solidus temperatures, which mark transitions between feeding stages. Figure 1 illustrates the relationships between feeding mechanisms and their associated solidification temperatures. The pouring and liquidus temperatures define liquid feeding. Mass feeding occurs between the liquidus and dendrite coherency temperatures. Interdendritic feeding takes place between the dendrite coherency and rigidity temperatures. Burst feeding occurs between the rigidity and solidus temperatures, and solid feeding happens below the solidus temperature.

In Figure 1, Tw represents the cooling curve measured near the mold wall, where heat extraction is faster due to direct contact with the mold. Tc represents the cooling curve measured in the center of the sample, where the cooling rate is slower. The temperature difference (Tw–Tc) thus reflects the thermal gradient between the mold wall and the sample center, which governs heat flow and solidification behavior, particularly the feeding mechanisms during solidification.

Thermal analysis (TA) has long been employed in the aluminum casting industry to determine characteristic solidification temperatures, such as the liquidus and solidus [10,11,12]. To identify additional temperatures, such as dendrite coherency and rigidity points, researchers like Bäckerud and others [5,6,7] have applied ΔT curves (Tw–Tc). By using the first derivative and delta T curves, cooling curve analysis enables the precise identification of the boundary temperatures that define the feeding regions. These boundaries can then be used to qualitatively and quantitatively evaluate the feeding regions. However, attempts to quantify these regions in the literature have been unsuccessful [8,9].

Dendrite coherency and rigidity temperatures were identified from the ΔT curve. The ΔT curve was calculated as a difference between the temperature measured close to the wall of the thermal analysis cup (Tw) and the temperature measured simultaneously in the center of the thermal analysis cup (Tc). All obtained values collected during trials were plotted as a function of time, and the first and second minima on such curves were used to determine the dendrite coherency point (first minimum) and rigidity temperature (second minimum). The determination of these two temperatures is well-explained in the available literature [5,11].

Several factors influence the feeding behavior of cast aluminum alloys, including chemical composition, solidification characteristics (e.g., freezing range, intermetallic compound formation), casting design (geometry), casting process parameters (pouring temperature, mold temperature, grain refinement, and melt modification), and casting conditions (mold coatings and gating design) [3,13,14,15,16,17]. Among these factors, the influence of alloy composition can be effectively evaluated using thermal analysis techniques.

The primary goal of this study was to examine the effect of varying silicon content, ranging from 5 to 9 wt% in increments of 2%, on the feeding behavior of cast Al-Si-Mg0.4 alloys. Previous studies [7,18,19] and foundry experience indicate that silicon significantly influences the feeding ability of hypoeutectic Al-Si alloys by affecting the solidification process and resulting microstructure. Increased silicon content generally improves the feeding ability by narrowing the freezing range and enhancing fluidity [17,20]. A narrower freezing range promotes more uniform solidification and better compensation for volume contraction during the solidification process.

Under equilibrium conditions, the solidification of hypoeutectic Al-Si-Mg alloys (where the Si content is below the eutectic composition, ~12.6 wt% Si) typically begins with the formation of primary α-Al dendrites. As α-Al dendrites grow, the surrounding liquid becomes enriched in silicon and magnesium, followed by the development of the eutectic Al-Si phase, when the temperature reaches the eutectic point of ~577 °C.

This study investigated the effect of Si content (5–9 wt.%, in 2 wt.% increments) on the solidification temperatures and feeding behavior of Al-Si-Mg0.4 alloys. It also examined whether higher Si levels contribute to shrinkage porosity in the as-cast structure, intending to improve casting quality and reduce defects. Thermal analysis (TA) was employed to conduct the experimental evaluation.

## 2. Materials and Methods

### 2.1. Materials and Melting Procedure

Three different Al–Si–Mg alloys with the chemical compositions presented in Table 1 were produced. Pure aluminum (commercial purity 99.7 wt.%, TRIMET Aluminium SE, Aluminiumallee 1, Essen, Germany), silicon (commercial purity 99.0 wt.%, HOESCH Metallurgie GmbH Neue Str. 21, Niederzier, Germany), and pure magnesium (commercial purity 99.9 wt.%, HOESCH Metallurgie GmbH Neue Str. 21, Niederzier, Germany) were used as input materials. The content of the major alloying element varied between 5.11–9.03 wt.% Si. The magnesium content was maintained at a constant level of almost 0.4 wt.%. The chemical compositions of the investigated alloys were determined using Optical Emission Spectroscopy (OES) (Type Spectrolab, SPECTRO Analytical Instruments GmbH, Boschstr. 10, Kleve, Germany). The alloys were melted in Nabertherm melting furnaces (Models K 1/10–KC 2/15) with a capacity of 10 kg (Zeller GmbH, Industriestr. 1, Hohenems, Austria). No grain refining and modifier agents were added to the melt. Degassing was not applied to any of the experiments.

### 2.2. TA Procedure

Samples with masses of approximately 200 ± 10 g were poured into stainless steel cups (height of 60 mm and diameter of 50 mm). Two calibrated thermocouples K-type (OMEGA Engineering, Daimlerstrasse 26, Deckenpfronn, Germany), with an accuracy of ±0.10 °C, were inserted into the melt, and temperatures between 750–400 °C were recorded. The data for the TA were collected using a high-speed National Instruments data acquisition system (Type NI cDAQ-9171, National Instrument, 11,500 N. Mopac Expwy, Austin, TX, USA) linked to a personal computer. The cooling conditions were kept constant during all experiments and were approximately 10 °C/min (i.e., 0.166 °C/s). The cooling rate was calculated as the ratio of the temperature difference between liquidus and solidus temperatures to the total solidification time between these two temperatures. Each TA trial was repeated two times. The solidification process was carried out under constant and controlled conditions, ensuring that the differences observed in feeding behavior were solely attributable to the silicon content.

**Figure 2:**
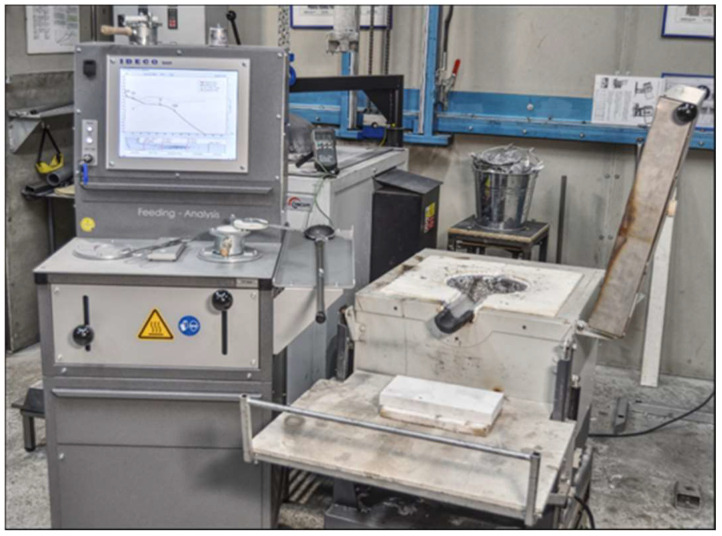
Layout of equipment during experiments.

Figure 2 shows a laboratory setup with the following equipment: Nabertherm electric resistance furnace and IDECO SA800SN thermal feeding analysis system (Ideco, AnFisserhook 3-5, Bocholt, Germany).

## 3. Results and Discussion

**Figure 3:**
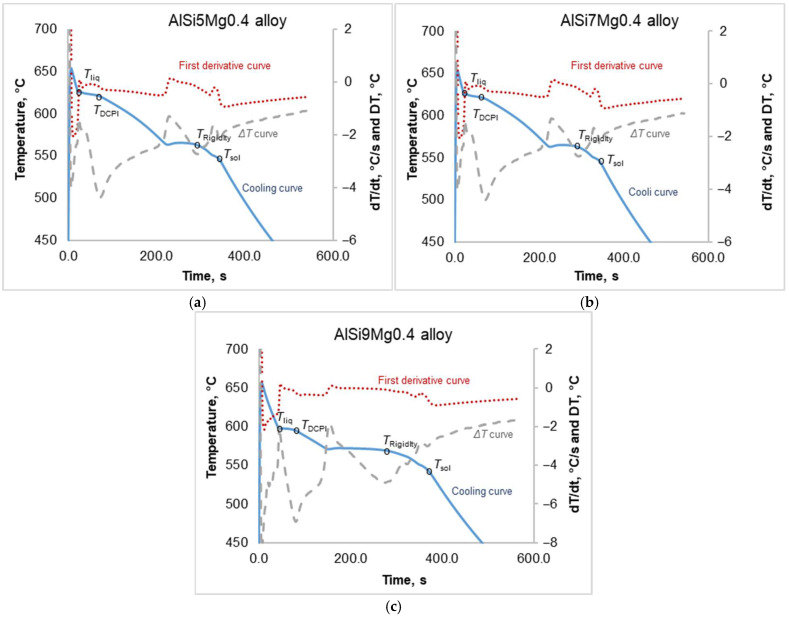
Cooling curves of hypoeutectic cast alloys: (**a**) AlSi5Mg0.4, (**b**) AlSi7Mg0.4, and (**c**) AlSi9Mg0.4.

Shrinkage porosity is a widespread defect in cast aluminum parts, resulting in significant scrap losses and restricting the use of cast components. This issue arises primarily from the alloy’s inadequate feeding ability during solidification. This study aimed to quantify the impact of alloying elements, particularly silicon, on the characteristic solidification temperatures of the alloys and the various feeding regions. Characteristic solidification temperatures were determined using cooling curve analysis. Figure 3 illustrates the influence of different silicon contents on the solidification paths of the investigated alloys: Al-Si5-Mg0.4, Al-Si7-Mg0.4, and Al-Si9-Mg0.4. As illustrated in Figure 3, the cooling curves (blue solid line), first derivative curves (red dotted line), and ΔT curves (grey dashed line) were utilized to identify the characteristic solidification temperatures of the investigated alloys. These temperatures were subsequently used to define the corresponding feeding regions. Additionally, specific solidification temperatures, such as the liquidus temperature, dendrite coherency temperature, rigidity, and solidus temperature, are precisely marked by circles for clarity. The liquidus and solidus temperatures were determined from the first derivative curves, where the liquidus corresponds to the initial sharp decrease in the cooling rate, and the solidus corresponds to the nearly constant cooling rate at the end of solidification. Dendrite coherency and rigidity temperatures were identified from the ΔT curve, corresponding to the first and second minima, respectively.

**Figure 4:**
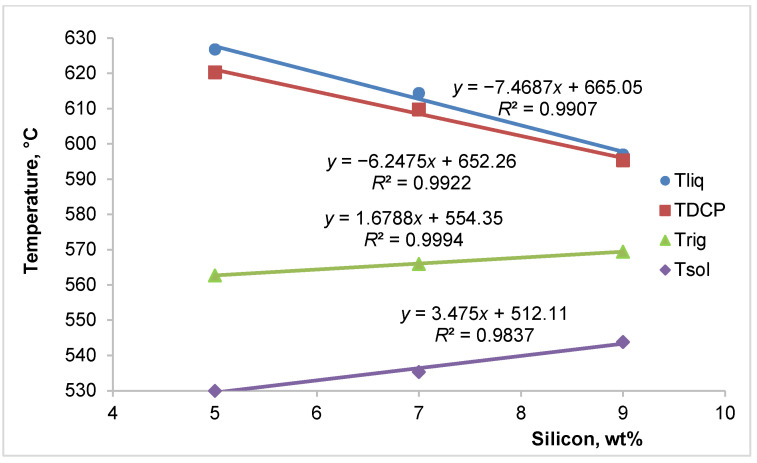
Impact of various contents of silicon on the characteristic solidification temperatures of the investigated alloys.

Figure 4 presents the influence of various silicon contents on the characteristic solidification temperatures of AlSi(5-7-9)Mg0.4 alloys. This figure shows that an increase in silicon content significantly lowered the liquidus and dendrite coherency temperatures while slightly increasing the rigidity and solidus temperatures. An increase in silicon content of up to 4 wt.% decreased the liquidus temperature by 29.8 °C and the dendrite coherency temperature by 25 °C.

According to the binary Al-Si phase diagram, 1 wt.% of silicon decreased the liquidus temperature by approximately 6.78 °C, meaning that the 4 wt.% difference in silicon content among the investigated alloys reduced the liquidus temperature by about 27 °C. Furthermore, based on the binary Al-Mg phase diagram, 0.4 wt.% magnesium lowered the liquidus temperature by an additional 2.2 °C (1 wt.% magnesium decreased the liquidus temperature by 5.5 °C). During solidification, primary α-Al dendrites formed first, rejecting silicon and magnesium into the remaining liquid. Increasing silicon content promoted the earlier onset of the eutectic reaction (α-Al + Si), thereby refining the dendritic network and reducing the interdendritic feeding interval. Magnesium remained mostly in solution until later stages, where it contributed to the formation of Mg_2_Si at the eutectic temperature. The combined effect of silicon and magnesium thus modified the solidification path and feeding effectiveness. Thus, the combined effect of silicon and magnesium reduced the liquidus temperature by 29.2 °C, which aligned closely with the cooling curve results, indicating a decrease of 29.8 °C.

Based on literature sources [20,21,22,23], silicon has a significant influence on the dendrite coherency temperature. Among foundry professionals, it is widely acknowledged that chemical composition substantially impacts dendrite size in addition to cooling rates. During the primary solidification of aluminum alloys, alloying elements are distributed unevenly between the solid and liquid phases. Excess solute is pushed out from the solidification interface into the melt, leading to solute enrichment between the formed dendrite arms. This solute supersaturation, or its associated constitutional undercooling, drives the growth of dendrites. Consequently, the spacing between the arms of α-aluminum dendrites expands to accommodate the increasing solute concentration. A higher concentration of alloying elements, such as silicon, promotes the formation of finer dendrites, thereby lowering the dendrite coherency temperature. According to the literature [20], increasing the silicon content in hypoeutectic Al-Si-Mg alloys from 5.1 to 8.9 wt.% lowers the dendrite coherency temperature by 24 °C. This result agrees well with the findings of this study. These changes significantly influenced the onset of mass and interdendritic feeding.

**Figure 5:**
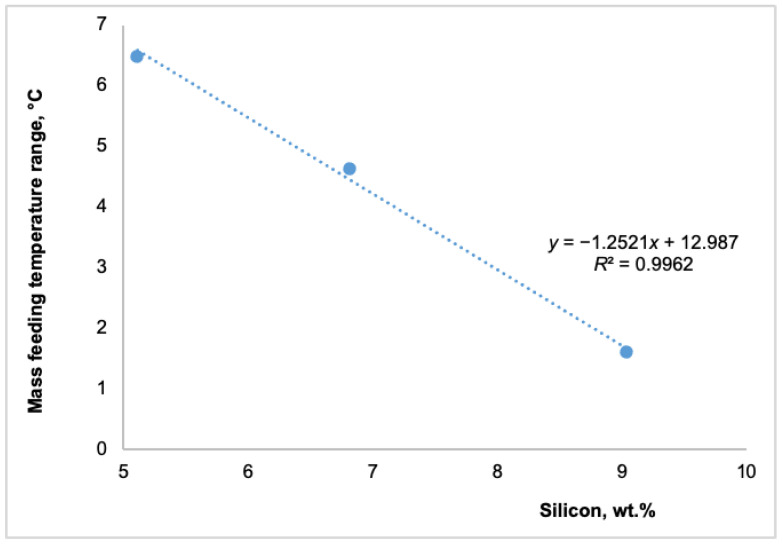
Impact of silicon on the mass-feeding region of Al-Si(5-9)-Mg0.4 alloys.

**Figure 6:**
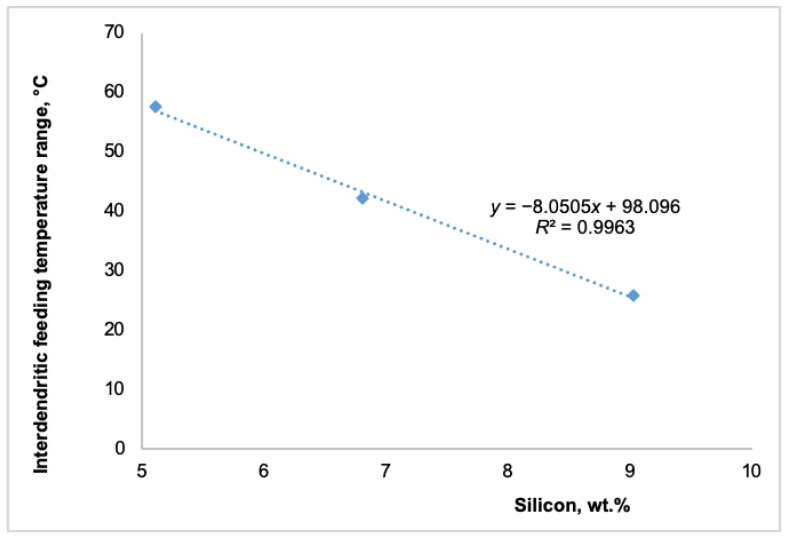
Impact of silicon on the interdendritic-feeding region of Al-Si(5-9)-Mg0.4 alloys.

**Figure 7:**
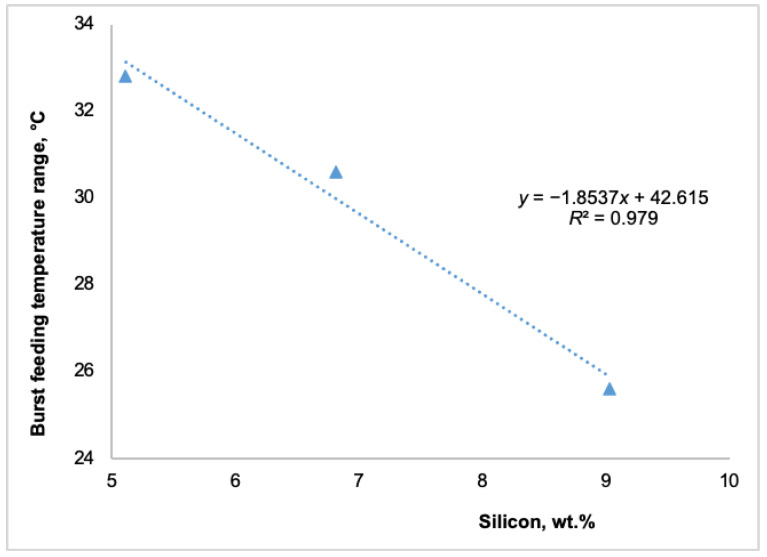
Impact of silicon on the burst-feeding region of Al-Si(5–9)-Mg0.4 alloys.

Simultaneously, as shown in Figure 4, an increase in silicon content raised the rigidity and solidus temperatures. A 4 wt.% increase in silicon content raised the rigidity temperature by 6.7 °C and the solidus temperature by almost 14 °C. These changes substantially impacted the various feeding regions. The subsequent figures illustrate the effect of different silicon contents on distinct feeding regions: the mass feeding region (Figure 5), the interdendritic feeding region (Figure 6), and the burst feeding region (Figure 7).

As shown in Figure 5, Figure 6 and Figure 7, adding silicon to Al-Si-Mg0.4 alloys significantly decreased their temperature feeding range. The available literature extensively documents that the solidification feeding behavior of cast aluminum alloys is intricately linked to their chemical composition [8,9,22,23]. These studies suggest that chemical composition plays a significant role in feeding behavior in addition to cooling rates.

Foundry experience has shown that higher silicon content reduces the feeding demand by narrowing the solidification range of hypoeutectic Al-Si-Mg alloys. This results in decreased levels of micro-shrinkage, enhancing the alloy’s stability. As illustrated in Figure 5, Figure 6 and Figure 7, adding up to 4 wt.% silicon to the Al-Si5-Mg0.4 alloy decreased the temperatures of all three feeding ranges as follows:

- Mass feeding decreased by 4.9 °C,
- Interdendritic feeding decreased by 31.7 °C, and
- Burst feeding decreased by 7.2 °C.

The shorter mass feeding region, which occurred at the beginning of solidification, had a limited impact on the overall solidification process. This was due to the smaller amount of primary α-aluminum solid phases, the relatively high solidification temperature, the wide active feeding path, and the lower viscosity at this stage. In contrast, the significant reduction in the temperature ranges of the interdendritic and burst feeding regions, approximately 32 °C and 7 °C, respectively, as shown in Figure 6 and Figure 7, can have a more substantial impact on producing sound cast parts. A shorter interdendritic feeding range results in a higher melt temperature, a lower solid fraction, and lower viscosity. Therefore, a 32 °C decrease in the interdendritic feeding temperature range is advantageous and could significantly reduce the formation of shrinkage porosity in as-cast products. Similarly, a shorter burst feeding temperature range can contribute to the production of sound cast parts free from shrinkage porosity. Being able to identify all characteristic solidification temperatures—liquidus, dendrite coherency, rigidity, and solidus—using the TA technique, which defines the five feeding regions, allows each area to be quantified using the equation proposed by Huber et al. [22].

**Figure 8:**
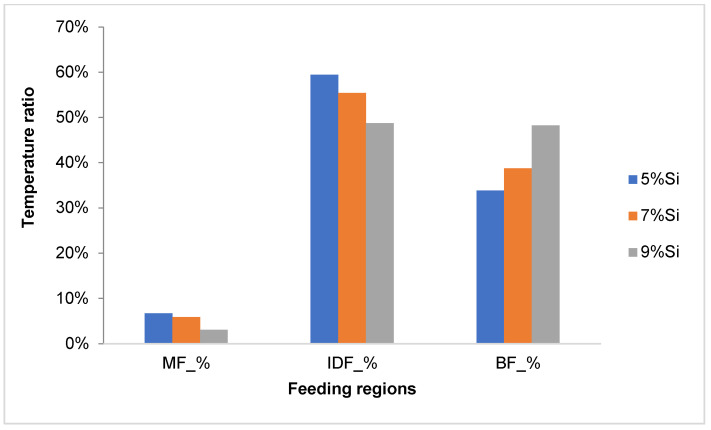
The impact of silicon on the temperature ratio of different feeding regions.

Using Equations (1)–(3) and calculating the corresponding temperature ratio for various feeding regions, the impact of silicon was quantified and is presented in Figure 8.

(1) $$MF=\frac{T_{lig}-T_{DCP}}{T_{lig}-T_{sol}}\cdot 100,$$

(2) $$IDF=\frac{T_{DCP}-T_{Rigidity}}{T_{lig}-T_{sol}}\cdot 100$$

(3) $$BF=\frac{T_{Rigidity}-T_{sol}}{T_{lig}-T_{sol}}\cdot 100$$

where:

MF—temperature ratio for mass feeding, %

IDF—temperature ratio for interdendritic feeding, %

BF—temperature ratio for burst feeding, %

$T_{lig}$—liquidus temperature, °C

$T_{DCP}$—dendrite coherency temperature, °C

$T_{Rigidity}$—rigidity temperature, °C

$T_{sol}$—solidus temperature, °C

As illustrated in Figure 8, silicon had a significant influence on the interdendritic and burst feeding regions. When the silicon content increased incrementally from 5.1 wt.% to 9.03 wt.%, the temperature ratio within the interdendritic feeding areas decreased by 10.7%, while the temperature ratio within the burst feeding regions increased by approximately 14.4%. Such variations in temperature ratios across the interdendritic and burst feeding regions could significantly impact the formation of shrinkage porosity in the resulting cast structures. Increasing the silicon content in cast hypoeutectic Al-Si(5,7,9)-Mg0.4 alloys generally enhanced their feeding ability up to a certain point due to silicon’s role in narrowing the freezing range and improving fluidity. A higher silicon content increased the eutectic phase in the microstructure, improving feeding by reducing the risk of shrinkage porosity. This occurs because a narrower freezing range promotes uniform solidification and better compensates for volume contraction during the solidification process. However, excessive silicon content (close to the eutectic concentration of 12.3 wt.%) can lead to coarse, brittle primary silicon phases, particularly if the alloy is not properly modified with elements such as strontium. These primary silicon particles may obstruct the flow of liquid metal during feeding, especially in complex or thin-walled castings, which can potentially increase the risk of porosity. To better understand the effect of silicon on shrinkage porosity formation, further analysis is required. These investigations should incorporate standard techniques, such as metallography, and advanced methodologies, including the “Sand Hourglass” test.

The observed shift, as seen in Figure 8, in the feeding mechanisms—quantified as a 10.7% decrease in the interdendritic-feeding ratio and a 14.4% increase in the burst-feeding ratio when the Si content increases from 5% to 9%—has significant implications for casting behavior. The decline in interdendritic feeding highlights a reduction in mushy-zone permeability, which can be attributed to the microstructural refinement induced by silicon. Higher Si levels reduce the secondary dendrite arm spacing (SDAS) and narrow the interdendritic channels, thereby restricting the flow of liquid metal through the semi-solid network. As a result, the capacity of the mushy zone to compensate for volumetric shrinkage is diminished, leading to a higher risk of shrinkage porosity and hot tearing.

At the same time, the higher burst-feeding ratio indicates a stronger reliance on the rapid pressure-driven redistribution of liquid metal. While this shift can partially offset shrinkage by enabling metal to reach isolated regions more quickly, it also amplifies the importance of controlling the initial flow conditions to avoid entrapped air, oxide films, and other gas-related defects.

In gravity casting, defects like interdendritic porosity appear more often because the metal solidifies slowly, creating wide interdendritic spaces. To avoid shrinkage, these spaces must be consistently filled with liquid metal from different feeding zones. On the other hand, in high-pressure die casting, the primary method for preventing defects is through burst feeding. Since the mold fills and solidifies very quickly, controlling the first flow of metal and the applied pressure is essential to reduce problems such as trapped air, oxide films, and other gas-related issues.

## 4. Conclusions

The present study investigated the influence of silicon content on the solidification behavior and feeding ability of hypoeutectic Al-Si-Mg alloys using the TA. The results clearly demonstrate that the silicon content has a systematic effect on the characteristic solidification temperatures, liquidus, dendrite coherency, rigidity, and solidus, and, consequently, on the extent of the different feeding regions.

An increase in silicon content from 5 to 9 wt.% significantly decreased both the liquidus and dendrite coherency temperatures, while slightly increasing the rigidity and solidus temperatures. These changes shortened the mass, interdendritic, and burst feeding ranges, with the most pronounced effect being a ~32 °C reduction in the interdendritic feeding region. From a foundry perspective, this reduction is highly beneficial: a shorter interdendritic feeding interval corresponds to higher liquid fraction, lower viscosity, and more effective melt feeding during critical stages of solidification. This reduces the likelihood of shrinkage porosity, which is one of the most common and detrimental defects in aluminum castings.

Quantitative evaluation further showed that silicon additions up to ~9 wt.% enhanced the feeding ability by narrowing the freezing range and promoting uniform solidification. This confirms industrial experience that higher silicon alloys are more resistant to shrinkage defects. However, the conclusions must also recognize that excessive silicon contents, approaching the eutectic composition (12.3 wt.%), may lead to the formation of coarse primary silicon particles. These phases can obstruct feeding and deteriorate mechanical properties unless proper grain refiners or modifiers are applied. Thus, there exists an optimal silicon window where feeding is maximized without negative side effects.

In summary, this work provides a clear quantitative link between silicon content, solidification temperatures, and feeding ability in hypoeutectic Al-Si-Mg alloys. By demonstrating how silicon narrows feeding ranges and improves melt compensation for shrinkage, the study delivers actionable insights for foundry practice. These findings can be directly applied by alloy designers and process engineers to improve casting soundness, reduce scrap rates, and optimize the use of Al-Si-Mg alloys in industrial production. It should be noted that the present findings are directly applicable to gravity casting and low-pressure die casting processes with moderate cooling rates. Extension to high pressure die casting (HPDC), where extremely rapid solidification (<1 s) and pressure assisted feeding mechanisms dominate, requires further investigation to validate the applicability of TA-based quantification under such conditions.

## Figures and Tables

**Table 1:** Chemical compositions of synthetic AlSi(5,7,9)Mg0.4 alloys.

Alloy	wt. %						
	Si	Mg	Cu	Mn	Cr	Ni	Zn
AlSi5Mg0.4	5.11	0.405	0.006	0.003	0.002	0.001	0.008
AlSi7Mg0.4	6.81	0.407	0.007	0.005	0.002	0.002	0.009
AlSi9Mg0.4	9.03	0.416	0.006	0.004	0.002	0.001	0.008

## Data Availability

The original contributions presented in this study are included in the article. Further inquiries can be directed to the corresponding author.
